# Classification systems for causes of stillbirth and neonatal death, 2009–2014: an assessment of alignment with characteristics for an effective global system

**DOI:** 10.1186/s12884-016-1040-7

**Published:** 2016-09-15

**Authors:** Susannah Hopkins Leisher, Zheyi Teoh, Hanna Reinebrant, Emma Allanson, Hannah Blencowe, Jan Jaap Erwich, J. Frederik Frøen, Jason Gardosi, Sanne Gordijn, A. Metin Gülmezoglu, Alexander E. P. Heazell, Fleurisca Korteweg, Joy Lawn, Elizabeth M. McClure, Robert Pattinson, Gordon C. S. Smith, Ӧzge Tunçalp, Aleena M. Wojcieszek, Vicki Flenady

**Affiliations:** 1 Mater Research Institute, The University of Queensland (MRI-UQ), Brisbane, Australia; 2 International Stillbirth Alliance, Millburn, USA; 3 Department of Reproductive Health and Research including UNDP/UNFPA/UNICEF/WHO/World Bank Special Programme of Research, Development and Research Training in Human Reproduction (HRP), World Health Organization, Geneva, Switzerland; 4 School of Women’s and Infants’ Health, Faculty of Medicine, Dentistry and Health Sciences, University of Western Australia, Perth, Australia; 5 London School of Hygiene & Tropical Medicine, London, UK; 6 University Medical Center Groningen, The University of Groningen, Groningen, The Netherlands; 7 Department of International Public Health, Norwegian Institute of Public Health, Oslo, Norway; 8 Center for Intervention Science for Maternal and Child Health, University of Bergen, Bergen, Norway; 9 Perinatal Institute, Birmingham, UK; 10 Maternal and Fetal Health Research Centre, University of Manchester, Manchester, UK; 11 St. Mary’s Hospital, Central Manchester University Hospitals NHS Foundation Trust, Manchester Academic Health Science Centre, Manchester, UK; 12 Department of Obstetrics and Gynaecology, Martini Hospital, Groningen, The Netherlands; 13 Research Triangle Institute, North Carolina, USA; 14 South Africa Medical Research Council Maternal and Infant Health Care Strategies Unit, University of Pretoria, Pretoria, South Africa; 15 NIHR Biomedical Research Centre & Department of Obstetrics & Gynaecology, Cambridge University, Cambridge, UK

**Keywords:** Stillbirth, Neonatal death, Perinatal death, Classification, Classification system, Cause

## Abstract

**Background:**

To reduce the burden of 5.3 million stillbirths and neonatal deaths annually, an understanding of causes of deaths is critical. A systematic review identified 81 systems for classification of causes of stillbirth (SB) and neonatal death (NND) between 2009 and 2014. The large number of systems hampers efforts to understand and prevent these deaths. This study aimed to assess the alignment of current classification systems with expert-identified characteristics for a globally effective classification system.

**Methods:**

Eighty-one classification systems were assessed for alignment with 17 characteristics previously identified through expert consensus as necessary for an effective global system. Data were extracted independently by two authors. Systems were assessed against each characteristic and weighted and unweighted scores assigned to each. Subgroup analyses were undertaken by system use, setting, type of death included and type of characteristic.

**Results:**

None of the 81 systems were aligned with more than 9 of the 17 characteristics; most (82 %) were aligned with four or fewer. On average, systems were aligned with 19 % of characteristics. The most aligned system (Frøen 2009-Codac) still had an unweighted score of only 9/17. Alignment with individual characteristics ranged from 0 to 49 %. Alignment was somewhat higher for widely used as compared to less used systems (22 % v 17 %), systems used only in high income countries as compared to only in low and middle income countries (20 % vs 16 %), and systems including both SB and NND (23 %) as compared to NND-only (15 %) and SB-only systems (13 %). Alignment was higher with characteristics assessing structure (23 %) than function (15 %).

**Conclusions:**

There is an unmet need for a system exhibiting all the characteristics of a globally effective system as defined by experts in the use of systems, as none of the 81 contemporary classification systems assessed was highly aligned with these characteristics. A particular concern in terms of global effectiveness is the lack of alignment with “ease of use” among all systems, including even the most-aligned. A system which meets the needs of users would have the potential to become the first truly globally effective classification system.

**Electronic supplementary material:**

The online version of this article (doi:10.1186/s12884-016-1040-7) contains supplementary material, which is available to authorized users.

## Background

Classification of the causes of the 5.3 million perinatal deaths (stillbirths and neonatal deaths) that occur each year is critical to reducing these deaths; it increases our understanding of underlying causes and enables comparison of causes within and between countries [1, 2]. In a related manuscript, we describe a systematic review which identified 81 classification systems for causes of stillbirth and neonatal death (in addition to the World Health Organization (WHO) International Classification of Diseases 10th revision (ICD-10)) that were created, modified, and/or used between 2009 and 2014, all with widely varying characteristics. Stated reasons for system development included the need to add features and missing categories, increase accuracy, reach new user groups, enable identification of underlying causes, and reduce the number of “unexplained” deaths [3].

The review found that alignment of systems with general principles of the ICD, the global standard for cause of death assignment and reporting, was somewhat limited, with just 21 % of systems using ICD codes. Systems were also found to have quite low coverage as measured by data from published reports between 2009 and 2014 showing numbers of deaths classified by each system, including in high-burden countries. The majority of systems were used only in the regions (high- or low/medium-income countries) where they had been developed.

Data produced by different systems are often incompatible, hampering efforts to increase understanding of the global burden of specific causes of perinatal deaths [4, 5]. In 2008, the WHO began work to rationalize the global approach to classification of causes of perinatal death. This approach, the ICD for Perinatal Mortality, or ICD-PM, is now in the testing phase [6]. As part of this effort, an iterative process to identify characteristics for an effective global classification system for causes of stillbirth (SB) and neonatal death (NND) was undertaken, and a global panel of experts in perinatal death classification identified 17 such characteristics (reported in this series; see Wojcieszek et al. [7]).

This is the second part of a two-part study. Part one was a systematic review of classification systems for causes of SB and NND created or used between 2009 and 2014; results are presented in this series [3].

The aim of the present study was to assess the alignment of identified classification systems against the expert-identified characteristics in order to inform work towards a globally effective approach for classification of causes of SB and NND.

## Methods

### Systems assessed

Eighty-one new, modified or used systems for SB and/or NND were identified through a systematic literature review reported in this series (see [3] for the methodology and results of this systematic review, including the PRISMA flowchart, and Additional file 1 for details of included systems). Throughout this paper, systems are referred to by first author and year of publication of the source document, e.g. “De Galan-Roosen 2002”, which is a standard way of labelling studies in systematic reviews, i.e. Cochrane. The many co-authors of some systems are named in the relevant citation.

### Outcome measures

1. Frequency of system alignment with individual characteristics for an effective global classification system;
2. Weighted and unweighted scores measuring system alignment against the set of all 17 characteristics.

The characteristics were those developed through expert consultation as reported by Wojcieszek et al. [7]. Ten characteristics related to systems’ structure, assessing comprehensiveness, relevance, validity, and sufficiency of detail for understanding cause of death. The remaining seven characteristics related to systems’ functioning, assessing reliability, accessibility, and value to users. In this paper, we assess alignment against the penultimate list of characteristics reported by Wojcieszek et al., which comprised eight structural characteristics and nine functional characteristics, as this was the format for which weights (percent agreement by the expert panel) were available.

Following are definitions of some terms used in this article:

- System: Any approach to classifying causes of neonatal deaths and/or stillbirths that was described by authors of included papers as a “system” or “approach”, and/or that included a clearly delineated list of causes separated from the data.
- Modified system: Any system that was created as a result of making changes to an existing system, where:
  - the system presented was described by the authors as a modification of an existing system, or
  - it was apparent that the system had been modified, despite the authors stating that the system was unchanged from its original form (e.g. different number of levels, number of categories at the top level, meaning of categories, etc.).
- New system: Any system that was created without modifying an existing system.
- Used system: A system that was used for any purpose (e.g. clinical, research) other than purely developmental (e.g. testing for reliability).
- Global system: Any system used to classify or estimate causes of stillbirths and neonatal deaths in all countries for which data is available.
- National system:
  - ∘ used by a national government for annual reporting of causes for the majority (>50%) of SB and/or NND nationwide, or
  - ∘ used by any research group (e.g. the United States Agency for International Development, USAID, or the United Nations Children’s Fund, UNICEF) to classify causes of death
    - ▪ as reported by Demographic and Health Surveys (DHS) in at least one year, where DHS data is assumed to be nationally representative, or
    - ▪ of the majority (>50%) of SB and/or NND that occur in a country in at least one year, or
  - ∘ otherwise stated to be a system developed on purpose for national government use.
- Widely used system: any system used to classify 1000+ deaths and/or in 2+ countries between 2009 and 2014.
- Level: Some systems may have a single “level” of causes and other systems may have several levels of causes, with the top level listing more general causes and each lower level listing sub-categories within a given general cause. For example, classifying the cause of a SB or NND in a system with multiple levels would mean that a set of causes, from most general (taken from the top level) to most specific (taken from the lowest level), would be selected, e.g. “congenital anomaly” from the top level and then more detail on that cause via assignation of a sub-category at the next level down, e.g. “trisomy 13”.

### Data collection and analysis

Rules were developed to extract variables to measure the 17 characteristics using information available in published reports (see Table 1 for a summary of rules, and Additional file 2 for greater detail).

**Table 1:** Summary of how alignment was assessed

	Characteristics	Weight	Variables used to assess alignment	Aligned if	Judgment of variable accuracy as a measure of alignment
	Structural characteristics				
1	A global system must use rules to ensure valid assignment of cause of death categories	.98	Rules available?	Yes	Strong
2	A global system must be able to work with all levels of data (from both low-income and high-income countries), including minimal levels	.98		Yes for all three variables	
			Used in both HIC and LMIC?		Strong
			Used with verbal autopsy?		Strong
			Used in >1 LMIC?		Weak
3	A global system must ensure cause of death categories are relevant in all settings	.96	Used in both HIC and LMIC?	Yes	Weak
4	A global system must require associated factors to be recorded and clearly distinguished from causes of death	.94		Yes for both variables below	
			Associated factors included?		Strong
			Distinguishes associated factors from causes?		Strong
5	A global system must distinguish between antepartum and intrapartum conditions	.90	Distinguishes IP from AP?	Yes	Strong
6	A global system should record the level of data available to assign the cause of death (e.g. verbal autopsy only, placental histology, autopsy, etc.)	.86	Records type of data used?	Yes	Strong
7	A global system must have multiple levels of causes of death, with a small number of main categories	.82		As below	
			Number of causes	≤10	Strong
			Number of levels	2+	Strong
8	A global system must include a sufficiently comprehensive list of categories to result in a low proportion of deaths classified as “other”	.80	% “other”	Max <20 %	Weak
	Functional characteristics				
9	A global system must be easy to use, and produce data that are easily understood and valued by users	1		As below	
			# deaths classified/# countries of use	500+ cases and/or 2+ countries	Weak
			Definitions available?	Yes	Weak
			Rules available?	Yes	Weak
			National?	Yes	Weak
10	A global system must have clear guidelines for use and definitions for all terms used	1		Yes for both variables below	
			Definitions available?		Strong
			Rules available?		Strong
11	A global system must produce data that can be used to inform strategies to prevent perinatal deaths	.96		As below	
			IP vs AP?	Yes	Weak
			% “other”	Max <20 %	Weak
			National?	Yes	Weak
12	A global system must require neonatal deaths to be clearly distinguished from stillbirths	.94		Yes for both variables below	
			Distinguishes SB and NND?		Strong
			Separate categories for SB and NND?		Strong
13	A global system must have high inter- and intra-rater reliability	.94	Reliability testing?	Yes; min ≥0.60	Strong
14	A global system must be available in different formats including inexpensive ehealth and mhealth options, and in multiple languages	.92		Yes for both variables below	
			E-format?		Strong
			>1 language?		Weak
15	A global system must allow easy access to the data by the end-users	.92	Accessible data?	Yes	Weak
16	A global system must incorporate both stillbirths and neonatal deaths	.86	Both SB and NND?	Yes	Strong
17	A global system must require the single most important factor leading to the death to be recorded	.86		As below	
			Hierarchical?	No or partially	Weak
			Only 1 cause allowed?	Yes	Strong
			Includes FGR/IUGR/SGA?	No	Strong

Each system was assessed for alignment with individual characteristics and categorized as either “aligned” or “not aligned”. Frequency of system alignment with individual characteristics was assessed. Overall system alignment with the full set of 17 characteristics was assessed using two measures: a weighted and an unweighted score. The unweighted score for a system was calculated by adding the total number of characteristics with which a system was aligned. The weighted score was equal to the total of the weights for each characteristic with which the system was aligned, where the weights represented the percentage of experts who had voted to include that characteristic, as reported by Wojcieszek et al. ([7]). Thus, if all experts agreed to include a characteristic, its weight was 1, and if 80 % agreed, its weight was 0.80. The maximum possible unweighted and weighted scores were 17 and 15.64, respectively.

Sensitivity to cut-offs for quantitative variables was assessed by reanalyzing system alignment at higher and lower cut-offs and comparing the resulting lists of most-aligned systems. Sensitivity analyses were also undertaken to determine the effect of excluding variables judged to measure a given characteristic less well (“weak” variables). For example, the variable recording the number of categories at the highest level of a system was judged to be particularly robust (“strong”) in measuring characteristic 7, which calls for systems to have a small number of main categories, as data extraction was straightforward. On the other hand, the variable recording whether a system was available in more than one language was judged to be less robust (“weak”) in measuring characteristic 14, since it was possible that we had missed systems in languages not commonly found in the databases searched for the systematic literature review. The maximum possible unweighted and weighted scores using “strong” variables only were 12 and 11, respectively.

Subgroup analyses were undertaken to explore differences in alignment according to: (i) type of death included (SB only, NND only, or both); (ii) systems that were widely vs less used (a widely used system was defined as any system used to classify 1000 or more deaths and/or used in two or more countries between 2009 and 2014; details presented in [3]); (iii) region of use according to World Bank country classification (HIC vs LMIC) [8]; and (iv) type of characteristic (functional vs structural). For the type of characteristic, mean unweighted scores for alignment of all systems with functional and structural characteristics were calculated (with maximum possible scores of 9 and 8, respectively).

Data were entered into in Stata/IC 12.1 for analysis of frequency distributions. System developers who are co-authors were excluded from data extraction and analysis.

## Results

### Overall alignment

The range of unweighted scores for system alignment with the 17 expert-identified characteristics for an effective global system was 0 to 9 out of a maximum possible score of 17, meaning that none of the 81 systems was aligned with more than 9 of these characteristics (see Table 2). Most systems (82 %) were aligned with four or fewer characteristics. The range of weighted scores for system alignment with the characteristics was 0 to 7.94 out of a maximum possible score of 15.64; by this measure, systems were aligned with 19 % of characteristics on average (equivalent to an average weighted score of 2.82).

**Table 2:** Weighted and unweighted scores measuring system alignment against expert-identified characteristics

	Score using all variables		Score using “strong” variables only	
	Unweighted	Weighted	Unweighted	Weighted
Maximum possible score	17	15.64	12	11.00
Froen 2009-Codac [9]	9	7.94	8	7.14
Korteweg 2006-Tulip [10]	7	6.20	6	5.40
Black 2010-CHERG [11]	6	5.50	3	2.82
Cole 1986 [12]	6	5.48	5	4.52
Flenady 2009-PSANZ-PDC [13]	6	5.50	5	4.54
Kotecha 2014-Wales [14]	6	5.42	4	3.70
Ujwala 2012 [15]	6	5.18	5	4.38
Chan 2004-PSANZ-NDC [23]	5	4.46	4	3.66
Kidanto 2009 [24]	5	4.38	5	4.38
Lawn 2006-CHERG [25]	5	4.72	4	3.82
Manning 2013-maternal & fetal-Ireland [26]	5	4.52	4	3.60
Pattinson 1989 [27]	5	4.58	4	3.78
Schmiegelow 2012 [28]	5	4.42	5	4.42
Varli 2008-Stockholm [29]	5	4.58	4	3.78
Wigglesworth 1980 [30]	5	4.46	3	2.70
Abdellatif 2013 [31]	4	3.34	3	2.54
CMACE 2010-maternal & fetal [32]	4	3.60	4	3.60
CMACE 2010-neonatal [32]	4	3.46	3	2.66
de Galan-Roosen 2002 [16]	4	3.48	4	3.48
Engmann 2012 [33]	4	3.46	3	2.66
Flenady 2009-PSANZ-NDC [13]	4	3.58	3	2.78
Gardosi 2014-MAIN^a^	4	3.52	4	3.52
Gordijn 2009 [18]	4	3.68	4	3.68
Khanum 2009 [34]	4	3.42	3	2.62
Kidron 2009 [35]	4	3.78	4	3.78
McClure 2015 [42]^b^	4	3.74	5	4.60
Mo-Suwan 2009 [36]	4	3.52	4	3.52
MRC 2002-PPIP-South Africa [37]	4	3.52	3	2.72
National Services Scotland 2013-neonatal [38]	4	3.40	2	1.68
NIPORT 2005-Bangladesh [39]	4	3.70	2	1.98
Shah 2011 [40]	4	3.60	4	3.66
Van Diem 2010 [41]	4	3.46	3	2.66
VanderWielen 2011-WiSSP [42]	4	3.60	4	3.60
Wood 2012 [43]	4	3.44	4	3.44
Basys 2014-Lithuania [44]	3	2.58	3	2.58
Chan 2004-PSANZ-PDC [23]	3	2.84	3	2.84
CMACE 2011-maternal & fetal [45]	3	2.62	3	2.62
Cole 1989-ICE [46]	3	2.84	3	2.84
De Reu 2009-Tulip mod. [47]	3	2.52	2	1.72
Froen 2009-simplified Codac [9]	3	2.54	3	2.54
Gardosi 2005-ReCoDe [48]	3	2.76	2	1.80
Glinianaia 2010 [49]	3	2.52	2	1.72
Hey 1986 [50]	3	2.84	4	3.70
Hinderaker 2003 [51]	3	2.52	2	1.72
Manandhar 2010 [52]	3	2.70	4	3.56
National Services Scotland 2013-obstetric [38]	3	2.64	2	1.72
Nausheen 2013 [53]	3	2.88	4	3.74
Nga 2012 [54]	3	2.60	3	2.66
SCRN WG 2011 [55]	3	2.70	3	2.70
Simpson 2010 [56]	3	2.48	2	1.68
Abha 2011 [57]	2	1.68	2	1.68
Aggarwal 2011 [58]	2	1.78	1	0.98
Aggarwal 2013 [59]	2	1.74	1	0.94
Black-2010-CHERG^c^ [11]	2	1.68	2	1.68
De Reu 2009-Cole mod. [47]	2	1.66	2	1.72
De Reu 2009-Wigglesworth mod. [47]	2	1.66	2	1.72
Dias e Silva 2013 [60]	2	1.68	2	1.68
Dudley 2010-INCODE [61]	2	1.80	3	2.72
Hey 1986-short form [50]	2	1.66	2	1.72
Khanal 2011 [62]	2	1.66	1	0.86
Lawn 2009-consistent classification for causes of stillbirth [63]	2	1.88	2	1.88
Lawn 2010 [36]	2	1.82	1	0.86
Lawn 2012 [64]	2	1.82	1	0.86
National Services Scotland 2013-FIGO [38]	2	1.72	2	1.72
Olamijulo 2011 [65]	2	1.72	2	1.72
Seaton 2012 [66]	2	1.66	1	0.86
Serena 2013-Wigglesworth mod. [67]	2	1.66	1	0.86
Winbo 1998-NICE [68]	2	1.66	2	1.72
Winter 2013-Rwanda [69]	2	1.66	1	0.86
Cunningham^d^ 1997 [70]	1	0.82	1	0.82
Freitas 2012 [71]	1	0.86	1	0.86
Gupta 2012-Bhutan [72]	1	0.80	0	0.00
Hama Diallo 2012 [73]	1	0.82	1	0.82
Jehan 2009^e^ [74]	1	0.80	0	0.00
Kruse 2014 [75]	1	0.80	1	0.86
Nabeel 2012 [76]	1	0.82	2	1.68
Public Health Agency of Canada 2008 [77]	1	0.86	1	0.86
Rocha 2011 78	1	0.82	1	0.82
Smith 2010 [79]	1	0.80	1	0.86
Serena 2013-ReCoDe mod. [67]	0	0.00	0	0.00
Wou 2014 [80]	0	0.00	0	0.00

^a^Personal communications, O. Tuncalp to V. Flenady, 7/21/2014 and 7/23/2014

^b^System was included via expert referral in 2014 and this paper was selected as the citation after its publication in 2015

^c^Two modifications of CHERG in Black 2010; this is found in his Webappendix 2

^d^System is described in the reference given, but originates from [81]

^e^PubMed citation is for Imtiaz; we use the family name Jehan to refer to this system

The most aligned of the 81 systems was Frøen 2009-Codac [9], with an unweighted score of 9 and a weighted score of 7.94. The next most aligned system was Korteweg 2006-Tulip [10], with an unweighted score of 7 and a weighted score of 6.20.

Five systems were next most aligned with the 17 expert-identified characteristics, according to both unweighted and weighted scores. These were Black 2010-CHERG [11], Cole 1986 [12], Flenady 2009-PSANZ-PDC [13], Kotecha 2014-Wales [14], and Ujwala 2012 [15]. All were aligned with 6 out of the 17 characteristics (i.e., an unweighted score of 6); they had weighted scores of 5.50, 5.48, 5.50, 5.42, and 5.18, respectively.

This group of seven most aligned systems included one global system and two national systems (used in Australia, New Zealand, and Wales). All but one (Black 2010-CHERG) were used for classifying both SB and NND. All but one (Cole 1986) were developed from 2006 onward. All but Kotecha 2014-Wales and Ujwala 2012 were “widely used” by our definition.

### Characteristics with greatest and least alignment

**Fig. 1:**
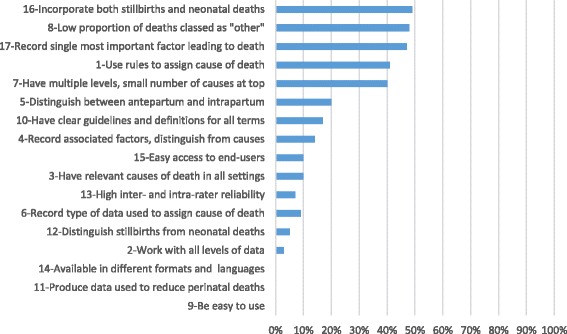
Percent of systems aligned with expert-identified characteristics for an effective global system. Note: Numbers in front of characteristics refer to sequence of characteristics in Table 1

System alignment with individual characteristics ranged from 0 to 49 % (see Table 3 and Fig. 1 for details). There were only five characteristics with which systems were highly aligned (i.e., 40 % or more systems aligned): (1) forty systems (49 %) were aligned with the requirement to incorporate both stillbirths and neonatal deaths, with LMIC-only systems somewhat less aligned than HIC-only systems (44 % v 56 %); (ii) just under half the systems were aligned with the requirement to produce a low proportion of deaths classified as “other”, with alignment particularly high for the NND-only systems as compared to the SB-only systems (65 % v 27 %); (iii) also just under half the systems were aligned with the requirement to record the single most important factor leading to death, with alignment of SB-only systems somewhat lower than for NND-only systems (33 % v 50 %); (iv) thirty-three systems (41 %) were aligned with the requirement to use rules for valid assignment of cause of death, a feature that was more common among widely used than less used systems (52 % v 35 %), HIC-only than LMIC-only systems (44 % v 28 %), and SB-only than NND-only systems (53 % v 35 %); and (v) thirty-two systems (40 %) were aligned with the requirement to have multiple levels and a small number of causes at the top level.

**Table 3:** System alignment with expert-identified characteristics for an effective global classification system for causes of stillbirth and neonatal death

	Characteristics	% consensus	% systems in alignment with each characteristic							
			All (81)	Widely used^a^ (27)	Less used (54)	Used in HIC only (36)	Used in LMIC only (32)	SB-only systems (15)	NND-only systems (26)	Combined systems (NND and SB) (40)
	Structural									
1	A global system must use rules to ensure valid assignment of cause of death categories.	98 %	41 %	52 %	35 %	44 %	28 %	53 %	35 %	40 %
2	A global system must be able to work with all levels of data (from both low-income and high-income countries), including minimal levels.	98 %	3 %	7 %	0 %	0 %	0 %	0 %	8 %	0 %
3	A global system must ensure cause of death categories are relevant in all settings.	96 %	10 %	30 %	0 %	0 %	0 %	7 %	15 %	8 %
4	A global system must require associated factors to be recorded and clearly distinguished from causes of death.	94 %	14 %	19 %	11 %	17 %	13 %	7 %	8 %	20 %
5	A global system must distinguish between antepartum and intrapartum conditions.	90 %	20 %	19 %	20 %	22 %	16 %	20 %	0 %	33 %
6	A global system should record the level of data available to assign the cause of death (e.g. verbal autopsy only, placental histology, autopsy, etc.).	86 %	9 %	19 %	4 %	19 %	0 %	7 %	4 %	13 %
7	A global system must have multiple levels of causes of death, with a small number of main categories.	82 %	40 %	33 %	43 %	33 %	44 %	33 %	42 %	40 %
8	A global system must include a sufficiently comprehensive list of categories to result in a low proportion of deaths classified as “other”.	80 %	48 %	52 %	46 %	53 %	53 %	27 %	65 %	45 %
	Functional									
9	A global system must be easy to use, and produce data that are easily understood and valued by users.	100 %	0 %	0 %	0 %	0 %	0 %	0 %	0 %	0 %
10	A global system must have clear guidelines for use and definitions for all terms used.	100 %	17 %	15 %	19 %	17 %	16 %	20 %	19 %	15 %
11	A global system must produce data that can be used to inform strategies to prevent perinatal deaths.	96 %	0 %	0 %	0 %	0 %	0 %	0 %	0 %	0 %
12	A global system must require neonatal deaths to be clearly distinguished from stillbirths.	94 %	5 %	7 %	4 %	0 %	9 %	0 %	0 %	10 %
13	A global system must have high inter- and intra-rater reliability.	94 %	7 %	11 %	6 %	8 %	6 %	7 %	0 %	13 %
14	A global system must be available in different formats including inexpensive ehealth and mhealth options, and in multiple languages.	92 %	0 %	0 %	0 %	0 %	0 %	0 %	0 %	0 %
15	A global system must allow easy access to the data by the end-users.	92 %	10 %	11 %	9 %	14 %	6 %	0 %	12 %	13 %
16	A global system must incorporate both stillbirths and neonatal deaths.	86 %	49 %	48 %	50 %	56 %	44 %	0 %	0 %	100 %
17	A global system must require the single most important factor leading to the death to be recorded.	86 %	47 %	52 %	44 %	50 %	41 %	33 %	50 %	50 %

^a^“Widely used”: systems used in more than one country and/or to classify 1000 or more deaths

Alignment was 10 % or lower for nine characteristics: (i) just eight of the 81 systems (10 %) were aligned with the requirement that systems use categories that are “relevant in all settings” (the exact characteristic is “A global system must ensure cause of death categories are relevant in all settings”), including 8 of the 27 widely used systems (30 %) and 4 of the 26 NND-only systems (15 %); (ii) eight systems were aligned with the requirement to allow end-users easy access to the data, including five of the 36 HIC-only systems and three of the 26 NND-only systems; (iii) seven systems (9 %) were aligned with the requirement to record the type of data used to assign cause of death, including seven of the 36 systems used only in HIC (19 %); (iv) six systems (7 %) were aligned with the requirement that systems have high reliability, including five of the 40 systems classifying both SB and NND; (v) four systems (5 %) were aligned with the requirement that systems distinguish NND from SB; (vi) two systems were aligned with the requirement that systems be able to work with data from LMIC as well as HIC settings; and (vii) no systems were aligned with the requirements that systems produce data that can be used to inform strategies to prevent death, be easy to use and produce easily understood data, and be accessible (available online and in multiple languages).

### Subgroup analyses

#### Alignment according to type of death classified

Alignment according to type of death classified (SB only, NND only, or both) was broadly similar to overall alignment (see Table 3). The 26 NND-only systems had an average unweighted score of 2.58, meaning they were aligned with an average of 15 % of the 17 characteristics; the 15 SB-only systems were aligned with 13 % of the 17 characteristics on average, and the 40 combined systems with 23 % (data not shown).

Alignment with the eight structural characteristics was generally similar for SB-only, NND-only and combined (SB and NND) systems, but different for the nine functional characteristics, with the 15 SB-only systems having an average unweighted score of just 0.60 (meaning they were aligned with just 0.60 of these characteristics on average) and the 26 NND-only systems aligned with just 0.81, whereas the 40 combined systems were aligned with 2.00 of these characteristics on average.

Alignment with individual characteristics also varied somewhat according to type of death classified. Other than characteristics requiring certain types of deaths to be included (e.g. the one requiring intrapartum and antepartm SB to be distinguished), alignment varied most strongly for the characteristic which requires systems to have a low proportion of deaths classified as “other”: four out of the 15 SB-only systems, or 27 %, and 17 out of the 26 NND-only systems, or 65 %, were aligned. Systems including both types of death were more aligned with the requirement to include associated factors (20 %, v 7 % for SB-only systems and 8 % for NND-only systems). NND-only systems were least aligned with the requirement to use rules for assigning cause of death (35 %, v 40 % for combined systems and 53 % for SB-only systems), while NND-only and combined systems were both more aligned with the requirement to record the single most important factor leading to death—50 %, as opposed to 33 % for SB-only systems.

#### Alignment of widely used systems

The 27 widely used systems were somewhat more aligned than the 54 less used systems with all 17 characteristics, with an average unweighted score of 3.74 (aligned with an average of 22 % of the characteristics) as compared to 2.91 (aligned with an average of 17 %). Widely used systems were also more aligned with the eight structural characteristics than less used systems, with an average unweighted score of 2.30 as compared to 1.59; the main differences related to characteristics requiring rules for use, globally relevant categories, and recording of the type of data used to assign cause of death. Widely and less used systems were similar in terms of alignment with the nine functional characteristics.

#### Alignment by region of use

Systems used only in HIC and only in LMIC had generally similar alignment with the 17 characteristics (with average unweighted scores of 3.33 and 2.75, representing 20 % and 16 % of the maximum possible score, respectively). Alignment was also similar for structural and functional characteristics considered separately, though HIC-only systems were slightly more aligned within each group: HIC-only systems were aligned with 24 % of the eight structural characteristics and 16 % of the nine functional characteristics; the figures for LMIC-only systems were 19 % and 14 %, respectively. Systems used only in HIC were more aligned with the characteristics requiring systems to use rules to assign cause of death and to record the type of data used to assign cause of death.

#### Alignment by type of characteristic

On average, systems had a mean unweighted score of 1.83 for alignment with the eight characteristics assessing systems’ structure (equivalent to alignment with 23 % of these characteristics) and 1.36 of the nine characteristics assessing systems’ functioning (equivalent to alignment with 15 % of these characteristics).

#### Sensitivity analysis

The results of sensitivity analyses (see Methods and Additional file 3 for details) show that Frøen 2009-Codac remained the most-aligned system even when restricting the alignment assessment to only the “strong” variables, with an unweighted score of 8 out of a maximum possible score of 12 (meaning that it was aligned with 67 % of characteristics measured by “strong” variables), and a weighted score of 7.14 out of a maximum possible 11 (aligned with 65 % of characteristics measured by “strong” variables when weighting was applied). Similarly, Korteweg 2006-Tulip remained the second-most-aligned system even with the restricted analysis, with an unweighted score of 6 and a weighted score of 5.40.

Three other systems were also among the highest scoring independently of whether weaker variables were included or not: Cole 1986, Flenady 2009-PSANZ-PDC, and Ujwala 2012, with unweighted scores using only “strong” variables of 5 for each of these systems, and weighted scores of 4.52, 4.54, and 4.38, respectively.

Results of sensitivity testing for different cut-offs for quantitative variables used to assess alignment with characteristics 7, 8 and 13 showed that the number of aligned systems was not very sensitive to the cut-offs assessed (see Table 1 for list of characteristics and Additional file 3 for details).

## Discussion

This study is the first to apply characteristics for an effective global classification system, as identified by an external panel of experts, to a set of classification systems for causes of SB and NND that were identified through a comprehensive, systematic literature review without language limits, and which included modifications as well as new systems. We found that classification systems for causes of stillbirth and neonatal death were overall poorly aligned with expert-identified characteristics; no system was aligned with more than 9 of 17 characteristics. This lack of alignment of current systems with the characteristics of an “ideal” classification system for causes of perinatal death may contribute to the ongoing development of new and modified systems at the rate of ten a year for the previous five years, possibly hindering the potential for widespread acceptance of one classification system.

Several researchers have previously assessed classification systems against various characteristics for an effective system. De Galan-Roosen 2002 assessed 12 systems, including four included in our study (the Wigglesworth 1980, Cole 1986, Hey 1986, and de Galan-Roosen 2002 itself), against seven characteristics, four of which are similar to our expert-identified characteristics (reliability, explanation of underlying cause, inclusion of both SB and NND, and the percent of “unclassifiable” deaths) [16]. Flenady 2009 assessed six systems, five of which are included in our study (Cole 1986, Flenady 2009-PSANZ-PDC, Gardosi 2005-ReCoDe, Korteweg 2006-Tulip and Frøen 2009-Codac) against three characteristics, two of which are included among our expert-identified characteristics (ease of use and reliability) [82]. Frøen 2009 assessed 11 systems, at least six of which were included in our study (versions of Aberdeen and Pattinson were also included but the version is unknown), against seven characteristics, four of which are included among our expert-identified characteristics (number of categories per level, whether underlying cause is identified, what type of data are required for use, and reliability) [17]. The previous most comprehensive review we are aware of, Gordijn, assessed 35 systems, of which we have included 12, against six characteristics, only one of which is included among the expert characteristics (number of causes per level) [18].

De Galan [16] found that their own system was most in alignment with the characteristics they considered, followed by the Hovatta system [19]; Flenady 2009 found that Frøen 2009-Codac, Flenady 2009-PSANZ-PDC and Gardosi 2005-ReCoDe performed best overall; and Frøen 2009 found that Flenady 2009-PSANZ-PDC and Frøen 2009-Codac were most in compliance with the characteristics reviewed, while Korteweg 2006-Tulip would require only modest modification (a new category for intrapartum) to become compliant. Gordijn stated that “each system [reviewed] has its own strengths and weaknesses”, and proposed combining existing systems to capitalize on their strengths so as to produce a new approach that would be well-aligned with key characteristics for an effective system.

A major difference between this study and prior reviews was our approach of assessing overall alignment of a comprehensively identified set of systems using a weighted scoring system against characteristics developed transparently by an external panel of experts. Despite this difference, we also identified Frøen 2009-Codac as the most aligned with expert characteristics for an effective global system, according to both unweighted and weighted scoring and regardless of whether we included only “strong” variables in the assessment or not. Four other systems were also consistently identified as among the most-aligned regardless of the scoring approach: Korteweg 2006-Tulip, which was consistently the second-most-aligned system, and Flenady 2009-PSANZ-PDC, Cole 1986, and Ujwala 2012. These results are similar to the findings of the Flenady and Frøen reviews [17, 82].

The concordance of these reviews may indicate underlying strengths of these systems, but must also be regarded in light of our finding of poor alignment even among the most aligned systems. We therefore suggest that rather than “best” systems, we have instead identified the most-aligned of a group that still lacks some essential features needed for effective global use. For instance, Frøen 2009-Codac, which we found to be the most-aligned system, and which was recently adopted by the UK for use in its national perinatal mortality surveillance, has shown a high proportion of stillbirths classified with “unknown” as the primary cause of death (47 % and 46 % from the first two annual reports in 2013 and 2014, respectively) [20, 21]. This high rate of “unknown” stillbirths using Codac in a high-income country has occurred despite education and training for the designated hospital-based staff who submit the data. However, disaggregation of the data (as the “unknown” category in Codac includes subcategories of both “unexplained” deaths despite thorough investigation, and “unknown” deaths with insufficient investigation or documentation) could help indicate the need for improved investigation of stillbirths as well as areas in need of strengthening within the system itself.

This example highlights the fact that while education and training for system implementation are necessary, they may not be sufficient to classify causes of perinatal death adequately. There remains a need for a system that is fully aligned with expert-identified characteristics for an effective global solution, notably including alignment with characteristics calling for the ability to work with all levels of data, from both HIC and LMIC settings, “ease of use”, and the production of data that “can be used to inform strategies to prevent perinatal death”.

It might be expected that a globally effective system would be aligned with the characteristics we found to have highest alignment among identified systems—hence, that it would provide rules for use, have multiple levels and a small number of categories at the top level, produce no more than 20 % of deaths classified as “other”, include both SB and NND, and record the single most important factor leading to death. Such a system would stand out from existing systems for also being aligned with the characteristics we found to have lowest alignment overall, in particular, the three characteristics absent from all systems (that systems should be easy to use and produce easily understandable data, produce data that can be used to inform strategies to prevent perinatal death, and be available in ehealth and mhealth options and in multiple languages). Having these features would strongly distinguish any new system from the rest.

Development of a globally effective system may also benefit from reference to systems that we identified as more aligned, despite their low alignment ratings overall. For instance, Frøen 2009-Codac was alone among the more aligned systems in providing a link for users to access data that are produced by the system. There are seven other systems we found which provide this access, one global and all the rest national systems. It may also be of interest to examine the characteristics of the national systems we found that are more aligned. In addition to being used nationally, these two systems (Kotecha 2014-Wales and Flenady 2009-PSANZ-PDC) were both aligned with two characteristics: they provided rules for use, and they included both SB and NND. A globally effective system might therefore stand apart from the large number of existing systems if it also bore these characteristics.

That combined systems (those incorporating both SB and NND) were somewhat more aligned than SB-only and NND-only systems may be a reflection of the weight placed upon this feature within the assessment methodology, with two characteristics dependent upon it (requiring SB to be distinguished from NND, and requiring inclusion of both types of death). An effective global system must incorporate both SB and NND. Given the somewhat greater alignment of the 27 widely used systems, it may also be of interest to note key features of these, which included identification of the single most important factor leading to death, greater availability of rules for use, definitions for some or all causes of death, and allowing associated factors to be recorded [3]. The slightly higher alignment of systems used only in HIC as compared to only in LMIC could point to a need for particularly careful implementation of a system intended to be globally effective, in order to identify and address any differences in functioning, acceptance, access, or interpretation across settings.

Given the finding of overall lower alignment with functional as compared to structural characteristics, attention should also be paid to ensuring a new system exhibits some of the key functional characteristics, including reliability (systems scored low on this more due to the lack of any reliability testing than to low Kappa scores) and accessibility (systems scored low on this due to lack of availability online and in multiple languages).

Another approach that may be of use to policy makers and public health officials in low-resource settings seeking to apply the results of this research would be to prioritize the characteristics and work toward alignment of their classification systems to the higher-priority ones first. During the process of identifying characteristics [7], panellists were not asked to rank them, rather, to indicate their level of agreement that a given characteristic was important for a globally effective system. Hence, each characteristic was judged on its own merit, not in conjunction with other characteristics. With an agreed cut-off of 80 % of more panellists stating “agree” or “strongly agree” with the characteristic’s importance for a globally effective system, 17 characteristics were ultimately selected. The percent agreement (shown in Table 1 as the weights for each characteristic) could be taken as a rough proxy for rank. The differences between characteristics are necessarily not very pronounced, since all had at least 80 % agreement. Yet still, some were less strongly supported than others. There are six characteristics with 96 % agreement or more, which could be a starting point for lower-resourced settings:

- A global system must be easy to use, and produce data that are easily understood and valued by users (agreed by 100 % of panellists)
- A global system must have clear guidelines for use and definitions for all terms used (agreed by 100 % of panellists)
- A global system must use rules to ensure valid assignment of cause of death categories (agreed by 98 % of panellists)
- A global system must be able to work with all levels of data (from both low-income and high-income countries), including minimal levels (agreed by 98 % of panellists)
- A global system must ensure cause of death categories are relevant in all settings (agreed by 96 % of panellists)
- A global system must produce data that can be used to inform strategies to prevent perinatal deaths (agreed by 96 % of panellists)

This study had some limitations. There was not a one-to-one correspondence between characteristics and the variables meant to measure these characteristics, and we relied on information available in published reports, which often lacked the detail required to measure characteristics precisely. This, along with the inherently more subjective nature of some characteristics (for instance, the characteristic requiring systems to produce data “that can be used to inform strategies to prevent perinatal deaths”), meant that some characteristics were found to be measured less accurately (designated as “weak” variables in Additional file 2) than others. However, the sensitivity analysis which excluded all “weak” variables from the assessment of alignment produced a similar list of most-aligned systems, indicating the methodology was not particularly sensitive to variables’ “strength”.

The number of deaths classified by national systems may have been underestimated due to retaining only the most recent paper between 2009 and 2014 that described a national system. This would have affected the assessment of alignment with the characteristic requiring systems to be easy to use and produce easily understandable data, as this relied in part on the number of deaths classified. However, this is unlikely to have affected overall results, as four other variables were also incorporated into the assessment of alignment for this characteristic (which was found to be 0 % for all systems).

The list of expert-identified characteristics did not include two characteristics relevant to the ICD-PM, namely whether ICD codes were used and whether both a maternal and a fetal/neonatal condition are required [22]. Both these characteristics were considered by the expert panel but ultimately did not receive 80 % or greater consensus [7]. However, the characteristic requiring systems to record associated factors and distinguish them clearly from causes of death may overlap with the concept of inclusion of both maternal and fetal/neonatal conditions. Data on this characteristic and the use of ICD codes are described in Leisher et al. 2016 in this series [3].

“Hierarchy”, meaning a set of rules forcing causes to be selected or rejected in a pre-determined order, was not included among the expert-identified characteristics. This is a common feature of systems (nearly one-third of systems we assessed were at least partially hierarchical), and is meant to assist in consistent assignment of cause of death when multiple conditions are present. However, along with two other variables, the “hierarchical” variable was used to assess alignment with the characteristic requiring the single most important factor leading to death to be recorded, with a value of “not hierarchical” or “partially hierarchical” indicating alignment. In recognition of the fact that there was no consensus on whether a globally effective system should be hierarchical [7], this variable was judged to be “weak”, and hence excluded in the sensitivity analysis.

## Conclusion

Despite the large number of classification systems recently used and/or developed (81), there remains an unmet need for a system that is aligned with expert-identified characteristics. To increase acceptance by potential users, ease of use and accessibility will be important, including availability online and in multiple languages, provision of links to data produced by the system, and education and training for potential users. A system including these features would have the potential to become the first truly globally effective classification system, making a critical contribution to the efforts of researchers, practitioners and policy makers in all countries to prevent the tragic loss of life—5.3 million stillbirths and neonatal deaths every year.

## Additional files

Additional file 1: 81 included systems and selected features. (DOCX 122 kb) Additional file 2: Variables used to assess system alignment with expert-identified characteristics for an effective global classification system for causes of stillbirth and neonatal death. (DOCX 57.8 kb) Additional file 3: Sensitivity analyses. (DOCX 46.3 kb)

## Abbreviations

AP: Antepartum
CHERG: Child health epidemiology reference group
CMACE: Centre for maternal and child enquiries
Codac: Causes of death and associated conditions
DHS: Demographic and health surveys
FGR: Fetal growth restriction
FIGO: International federation of gynaecology and obstetrics
HIC: High-income countries
ICD: International classification of diseases
ICD-PM: International classification of diseases for perinatal mortality
ICE: International collaborative effort
INCODE: Initial causes of fetal death
IP: Intrapartum
IUGR: Intrauterine growth restriction
LMIC: Low- and middle-income countries
MAIN: The maternal, antenatal, intrapartum & neonatal classification system for perinatal deaths
MRC: Medical research council
NICE: Neonatal and intrauterine death classification according to etiology
NIPORT: National institute of population research and training
NND: Neonatal death
PPIP: Perinatal problem identification programme
PSANZ-NDC: Perinatal Society of Australia and New Zealand Neonatal Death Classification
PSANZ-PDC: Perinatal Society of Australia and New Zealand Perinatal Death Classification
ReCoDe: Relevant condition at death
SB: Stillbirth
SCRN WG: The stillbirth collaborative research network working group
SGA: Small for gestational age
WHO: World Health Organization
WiSSP: Wisconsin stillbirth service program
