# Supplementary material for: Classification systems for causes of stillbirth and neonatal death, 2009–2014: an assessment of alignment with characteristics for an effective global system
Source: BMC Pregnancy Childbirth. 2016 Sep 15;16:269. doi: 10.1186/s12884-016-1040-7 (PMC5025539; doi:10.1186/s12884-016-1040-7)
Supplement: Additional file 1: — 81 included systems and selected features. (DOCX 122 kb) [file 12884_2016_1040_MOESM1_ESM.docx]

## Additional file 1

### 81 included systems and selected features

| **First author, year** | **No. of included systems in paper** | **Name(s) of new or modified system(s) (if any)** | **Used to classify >1000 deaths or in >1 country (“widely used”)?** | **National system?** | **Used in HIC, LMIC, or both?** |
| --- | --- | --- | --- | --- | --- |
| Abdellatif 2013 [[1](#_ENREF_2_1)] | 1 |  |  |  | HIC |
| Abha 2011 [[2](#_ENREF_2_2)] | 1 |  |  |  | LMIC |
| Aggarwal 2011 [[3](#_ENREF_2_3)] | 1 |  |  |  | LMIC |
| Aggarwal 2013 [[4](#_ENREF_2_4)] | 1 |  |  |  | LMIC |
| Basys 2014 [[5](#_ENREF_2_5)] | 1 |  |  | Yes | HIC |
| Black 2010 [[6](#_ENREF_2_6)] | 2 | CHERG (2 mods.) | Yes |  | Both (first mod.); unused for second mod. |
| Chan 2004 [[7](#_ENREF_2_7)] | 2 | PSANZ-NDC, PSANZ-PDC |  |  | HIC |
| CMACE 2010 [[8](#_ENREF_2_8)] | 2 | Maternal & fetal, neonatal | Yes (both) | Yes (both) | HIC |
| CMACE 2011 [[9](#_ENREF_2_9)] | 1 |  | Yes | Yes | HIC |
| Cole 1986 [[10](#_ENREF_2_10)] | 1 |  | Yes |  | Both |
| Cole 1989 [[11](#_ENREF_2_11)] | 1 | ICE | Yes |  | HIC |
| Cunningham 1997^a^ [[12](#_ENREF_2_12)] | 1 |  |  |  | LMIC |
| De Galan-Roosen 2002 [[13](#_ENREF_2_13)] | 1 | Fundamental Classification System |  |  | LMIC |
| De Reu 2009 [[14](#_ENREF_2_14)] | 3 | Wigglesworth, Cole 1986, and Tulip mods. |  |  | HIC |
| Dias e Silva 2013 [[15](#_ENREF_2_15)] | 1 | Brazilian list of avoidable deaths |  | Yes | LMIC |
| Dudley 2010 [[16](#_ENREF_2_16)] | 1 | INCODE | Yes |  | HIC |
| Engmann 2012 [[17](#_ENREF_2_17)] | 1 |  | Yes |  | LMIC |
| Flenady 2009 [[18](#_ENREF_2_18)] | 2 | PSANZ-PDC, PSANZ-NDC | Yes (both) | Yes (both) | Both (PDC), HIC (NDC) |
| Freitas 2012 [[19](#_ENREF_2_19)] | 1 |  |  |  | LMIC |
| Froen 2009 [[20](#_ENREF_2_20)] | 2 | Codac, Simplified Codac | Yes (Codac only) |  | HIC (Codac); unused for simplified Codac |
| Gardosi 2005 [[21](#_ENREF_2_21)] | 1 | ReCoDe | Yes |  | Both |
| Gardosi 2014^b^ | 1 | MAIN |  |  | Unused |
| Glinianaia 2010 [[22](#_ENREF_2_22)] | 1 |  |  |  | HIC |
| Gordijn 2009 [[23](#_ENREF_2_23)] | 1 | Multilayered approach |  |  | HIC |
| Gupta 2012 [[24](#_ENREF_2_24)] | 1 |  |  | Yes | LMIC |
| Hama Diallo 2012 [[25](#_ENREF_2_25)] | 1 |  |  |  | LMIC |
| Hey 1986 [[26](#_ENREF_2_26)] | 2 | Fetal and Neonatal Factors, short classification |  |  | LMIC (Fetal); HIC (short) |
| Hinderaker 2003 [[27](#_ENREF_2_27)] | 1 |  |  |  | LMIC |
| Jehan 2009^c^ [[28](#_ENREF_2_28)] | 1 |  |  |  | LMIC |
| Khanal 2011 [[29](#_ENREF_2_29)] | 1 |  |  |  | LMIC |
| Khanum 2009 [[30](#_ENREF_2_30)] | 1 |  |  |  | LMIC |
| Kidanto 2009 [[31](#_ENREF_2_31)] | 1 |  |  |  | LMIC |
| Kidron 2009 [[32](#_ENREF_2_32)] | 1 |  |  |  | HIC |
| Korteweg 2006 [[33](#_ENREF_2_33)] | 1 | Tulip | Yes |  | HIC |
| Kotecha 2014 [[34](#_ENREF_2_34)] | 1 | Clinico-pathological |  | Yes | HIC |
| Kruse 2014 [[35](#_ENREF_2_35)] | 1 |  |  |  | LMIC |
| Lawn 2006 [[36](#_ENREF_2_36)] | 1 | CHERG | Yes |  | Both |
| Lawn 2009 [[37](#_ENREF_2_37)] | 1 | Consistent Classification for Causes of Stillbirth |  |  | Unused |
| Lawn 2010 [[38](#_ENREF_2_38)] | 1 |  | Yes |  | Both |
| Lawn 2012 [[39](#_ENREF_2_39)] | 1 |  | Yes |  | Both |
| Manandhar 2010 [[40](#_ENREF_2_40)] | 1 |  | Yes |  | LMIC |
| Manning 2013 [[41](#_ENREF_2_41)] | 1 | Maternal & fetal |  | Yes | HIC |
| McClure 2014^d^ [[42](#_ENREF_2_42)] | 1 | Global Network |  |  | Unused |
| Mo-Suwan 2009 [[43](#_ENREF_2_43)] | 1 |  |  |  | LMIC |
| MRC 2002 [[44](#_ENREF_2_44)] | 1 | PPIP | Yes | Yes | LMIC |
| Nabeel 2012 [[45](#_ENREF_2_45)] | 1 |  |  |  | LMIC |
| National Services Scotland 2013 [[46](#_ENREF_2_46)] | 3 | Obstetric, FIGO, neonatal | Yes (FIGO only) | Yes (all) | HIC |
| Nausheen 2013 [[47](#_ENREF_2_47)] | 1 |  |  |  | LMIC |
| Nga 2012 [[48](#_ENREF_2_48)] | 1 |  |  |  | LMIC |
| NIPORT 2005 [[49](#_ENREF_2_49)] | 1 |  |  | Yes | LMIC |
| Olamijulo 2011 [[50](#_ENREF_2_50)] | 1 |  |  |  | LMIC |
| Pattinson 1989 [[51](#_ENREF_2_51)] | 1 |  |  |  | LMIC |
| Public Health Agency of Canada 2008 [[52](#_ENREF_2_52)] | 1 |  |  | Yes | HIC |
| Rocha 2011 [[53](#_ENREF_2_53)] | 1 |  | Yes |  | LMIC |
| Schmiegelow 2012 [[54](#_ENREF_2_54)] | 1 |  |  |  | LMIC |
| Seaton 2012 [[55](#_ENREF_2_55)] | 1 |  | Yes |  | HIC |
| Serena 2013 [[56](#_ENREF_2_56)] | 2 | Aberdeen and ReCoDe mods. |  |  | HIC |
| Shah 2011 [[57](#_ENREF_2_57)] | 1 |  |  |  | LMIC |
| Simpson 2010 [[58](#_ENREF_2_58)] | 1 |  |  |  | HIC |
| Smith 2010 [[59](#_ENREF_2_59)] | 1 |  | Yes |  | HIC |
| The Stillbirth Collaborative Research Network Writing Group 2011 [[60](#_ENREF_2_60)] | 1 |  |  |  | HIC |
| Ujwala 2012 [[61](#_ENREF_2_61)] | 1 |  |  |  | LMIC |
| VanderWielen 2011 [[62](#_ENREF_2_62)] | 1 | WiSSP |  |  | HIC |
| Van Diem 2010 [[63](#_ENREF_2_63)] | 1 |  |  |  | HIC |
| Varli 2008 [[64](#_ENREF_2_64)] | 1 | Stockholm | Yes |  | HIC |
| Wigglesworth 1980 [[65](#_ENREF_2_65)] | 1 | Wigglesworth | Yes |  | Both |
| Winbo 1998 [[66](#_ENREF_2_66)] | 1 | NICE | Yes |  | LMIC |
| Winter 2013 [[67](#_ENREF_2_67)] | 1 |  | Yes | Yes | LMIC |
| Wood 2012 [[68](#_ENREF_2_68)] | 1 |  | Yes |  | HIC |
| Wou 2014 [[69](#_ENREF_2_69)] | 1 |  |  |  | HIC |

^a^ System is described in the reference given, but originates from [[70](#_ENREF_2_70)].

^b^ Personal communications, O. Tuncalp to V. Flenady, 7/21/2014 and 7/23/2014.

^c^ PubMed citation is for Imtiaz; we use the family name Jehan to refer to this system.

^d^ System was included via expert referral in 2014 and this paper was selected as the citation after its publication in 2015.

Mod./Mods.: modification/modifications.

**References**

1. Abdellatif M, Al-Battashi A, Ahmed M, Bataclan MF, Khan AA, Al-Maniri A. The patterns and causes of neonatal mortality at a tertiary hospital in Oman. Oman Med J. 2013;28(6):422-6. doi:<http://dx.doi.org/10.5001/omj.2013.119>.

2. Abha S, Alpana T. Re. Co. De.: A better classification for determination of still births. Journal of Obstetrics and Gynecology of India. 2011;61(6):656-8.

3. Aggarwal AK, Jain V, Kumar R. Validity of verbal autopsy for ascertaining the causes of stillbirth. Bull World Health Organ. 2011;89(1):31-40. doi:10.2471/BLT.10.076828.

4. Aggarwal AK, Kumar P, Pandit S, Kumar R. Accuracy of WHO verbal autopsy tool in determining major causes of neonatal deaths in India. PLoS ONE [Electronic Resource]. 2013;8(1):e54865.

5. Basys V, Drazdienë N, Vezbergienë N, Isakova J. Gimimø medicininiai duomenys [Medical data of Births 2013]. Vilnius. Institute of Hygiene Health Information Centre, Vilnius University Medical Faculty, Vilnius University, Centre of Neonatology; 2014.

6. Black RE, Cousens S, Johnson HL, Lawn JE, Rudan I, Bassani DG et al. Global, regional, and national causes of child mortality in 2008: a systematic analysis. Lancet. 2010;375(9730):1969-87. doi:<http://dx.doi.org/10.1016/S0140-6736(10)60549-1>.

7. Chan A, King JF, Flenady V, Haslam RH, Tudehope DI. Classification of perinatal deaths: development of the Australian and New Zealand classifications. J Paediatr Child Health. 2004;40(7):340-7. doi:10.1111/j.1440-1754.2004.00398.x.

8. Centre for Maternal and Child Enquiries (CMACE). Perinatal Mortality 2008: United Kingdom. London. CMACE; 2010.

9. Centre for Maternal and Child Enquiries (CMACE). Perinatal Mortality 2009: United Kingdom. London. CMACE; 2011.

10. Cole SK, Hey EN, Thomson AM. Classifying perinatal death: an obstetric approach. Br J Obstet Gynaecol. 1986;93(12):1204-12.

11. Cole S, Hartford RB, Bergsjo P, McCarthy B. International collaborative effort (ICE) on birth weight, plurality, perinatal, and infant mortality. III: A method of grouping underlying causes of infant death to aid international comparisons. Acta Obstet Gynecol Scand. 1989;68(2):113-7.

12. Cunningham F LK, Bloom SL, Hauth JC, Rouse DJ, Spong CY, editor. Williams Obstetrics. 23rd ed. New York, NY: McGraw-Hill; 2010.

13. de Galan-Roosen AE, Kuijpers JC, van der Straaten PJ, Merkus JM. Fundamental classification of perinatal death. Validation of a new classification system of perinatal death. Eur J Obstet Gynecol Reprod Biol. 2002;103(1):30-6.

14. De Reu P, Van Diem M, Eskes M, Oosterbaan H, Smits L, Merkus H et al. The Dutch Perinatal Audit Project: a feasibility study for nationwide perinatal audit in the Netherlands. Acta Obstet Gynecol Scand. 2009;88(11):1201-8. doi:<http://dx.doi.org/10.3109/00016340903280990>.

15. Dias e Silva CMC, Gomes KRO, Rocha OAMS, de Almeida IMLM, Neto JMM. Validity and reliability of data and avoidability of the underlying cause of neonatal deaths in the intensive care unit of the North-Northeast Perinatal Care Network [Validade, confiabilidade e evitabilidade da causa basica dos obitos neonatais ocorridos em unidade de cuidados intensivos da Rede Norte-Nordeste de Saude Perinatal]. Cad Saude Publica. 2013;29(3):547-56.

16. Dudley DJ, Goldenberg R, Conway D, Silver RM, Saade GR, Varner MW et al. A new system for determining the causes of stillbirth. Obstet Gynecol. 2010;116(2 PART 1):254-60.

17. Engmann C, Garces A, Jehan I, Ditekemena J, Phiri M, Mazariegos M et al. Causes of community stillbirths and early neonatal deaths in low-income countries using verbal autopsy: an International, Multicenter Study. J Perinatol. 2012;32(8):585-92.

18. Flenady V, King J, Charles A, Gardener G, Ellwood D, Day K et al. PSANZ Clinical Practice Guideline for Perinatal Mortality. 2009.

19. Freitas BAC, Goncalves MR, Ribeiro RdCL. Infant mortality according to preventable causes and components - Vicosa-MG, 1998-2010. [Portuguese]. Pediatria Moderna. 2012;48(6):237-45.

20. Froen JF, Pinar H, Flenady V, Bahrin S, Charles A, Chauke L et al. Causes of death and associated conditions (Codac) - a utilitarian approach to the classification of perinatal deaths. BMC Pregnancy Childbirth. 2009;9(22). DOI: 10.1186/1471-2393-9-22.

21. Gardosi J, Kady SM, McGeown P, Francis A, Tonks A. Classification of stillbirth by relevant condition at death (ReCoDe): population based cohort study. 2005;331(7525):1113-7. doi:10.1136/bmj.38629.587639.7C.

22. Glinianaia SV, Rankin J, Pearce MS, Parker L, Pless-Mulloli T. Stillbirth and infant mortality in singletons by cause of death, birthweight, gestational age and birthweight-for-gestation, Newcastle upon Tyne 1961-2000. Paediatr Perinat Epidemiol. 2010;24(4):331-42. doi:<http://dx.doi.org/10.1111/j.1365-3016.2010.01119.x>.

23. Gordijn SJ, Korteweg FJ, Erwich JJHM, Holm JP, van Diem MT, Bergman KA et al. A multilayered approach for the analysis of perinatal mortality using different classification systems. Eur J Obstet Gynecol Reprod Biol. 2009;144(2):99-104.

24. Gupta SS. Identification of causes of under-five deaths in health facilities in Bhutan Ministry of Health of the Royal Government of Bhutan 2012.

25. Hama Diallo A, Meda N, Sommerfelt H, Traore GS, Cousens S, Tylleskar T et al. The high burden of infant deaths in rural Burkina Faso: a prospective community-based cohort study. BMC Public Health. 2012;12:739.

26. Hey EN, Lloyd DJ, Wigglesworth JS. Classifying perinatal death: fetal and neonatal factors. Br J Obstet Gynaecol. 1986;93(12):1213-23.

27. Hinderaker SG, Olsen BE, Bergsjo PB, Gasheka P, Lie RT, Havnen J et al. Avoidable stillbirths and neonatal deaths in rural Tanzania. BJOG. 2003;110(6):616-23.

28. Imtiaz J, Harris H, Sohail S, Amna Z, Naushaba M, Omrana P et al. Neonatal mortality, risk factors and causes: a prospective population-based cohort study in urban Pakistan. Bull World Health Organ. 2009;87(2):130-8. doi:<http://dx.doi.org/10.2471/BLT.08.050963>.

29. Khanal S, GC VS, Dawson P, Houston R. Verbal autopsy to ascertain causes of neonatal deaths in a community setting: a study from Morang, Nepal. JNMA, Journal of the Nepal Medical Association. 2011;51(181):21-7.

30. Khanum F. Perinatal mortality-one year analysis at tertiary care hospital of Peshawar. Journal of Postgraduate Medical Institute. 2009;23(3):267-71.

31. Kidanto HL, Mogren I, van Roosmalen J, Thomas AN, Massawe SN, Nystrom L et al. Introduction of a qualitative perinatal audit at Muhimbili National Hospital, Dar es Salaam, Tanzania. BMC Pregnancy Childbirth. 2009;9:45.

32. Kidron D, Bernheim J, Aviram R. Placental findings contributing to fetal death, a study of 120 stillbirths between 23 and 40 weeks gestation. Placenta. 2009;30(8):700-4.

33. Korteweg FJ, Gordijn SJ, Timmer A, Erwich JJ, Bergman KA, Bouman K et al. The Tulip classification of perinatal mortality: introduction and multidisciplinary inter-rater agreement. BJOG. 2006;113(4):393-401. doi:10.1111/j.1471-0528.2006.00881.x.

34. Kotecha S, Kotecha S, Rolfe K, Barton E, John N, Lloyd M et al. All Wales Perinatal Survey Annual Report 2013 Cardiff, Wales; 2014.

35. Kruse AY, Phuong CN, Ho BT, Stensballe LG, Pedersen FK, Greisen G. Identification of important and potentially avoidable risk factors in a prospective audit study of neonatal deaths in a paediatric hospital in Vietnam. Acta Paediatr. 2014;103(2):139-44. doi:10.1111/apa.12423 [doi].

36. Lawn JE, Wilczynska-Ketende K, Cousens SN. Estimating the causes of 4 million neonatal deaths in the year 2000. Int J Epidemiol. 2006;35(3):706-18. doi:10.1093/ije/dyl043.

37. Lawn JE, Yakoob MY, Haws RA, Tanya S, Darmstadt GL, Bhutta ZA. 3.2 million stillbirths: epidemiology and overview of the evidence review. (Special Issue: The global picture and evidence-based solutions.). BMC Pregnancy Childbirth. 2009;9(Suppl. 1). doi:<http://dx.doi.org/10.1186/1471-2393-9-S1-S2>.

38. Lawn JE, Kerber K, Enweronu-Laryea C, Cousens S. 3.6 Million neonatal deaths - what is progressing and what is not? (Special Issue: Global perinatal health.). Semin Perinatol. 2010;34(6):371-86. doi:<http://dx.doi.org/10.1053/j.semperi.2010.09.011>.

39. Lawn JE, Kinney MV, Black RE, Pitt C, Cousens S, Kerber K et al. Newborn survival: a multi-country analysis of a decade of change. (Special Issue: A decade of change for newborn survival, policy and programmes (2000-2010): A multi-country evaluation of progress towards scale.). Health Policy Plan. 2012;27(Suppl. 3). doi:<http://dx.doi.org/10.1093/heapol/czs053>.

40. Manandhar SR, Ojha A, Manandhar DS, Shrestha B, Shrestha D, Saville N et al. Causes of stillbirths and neonatal deaths in Dhanusha district, Nepal: a verbal autopsy study. Kathmandu University Medical Journal. 2010;8(1):62-72.

41. Manning E, Corcoran P, Meaney S, Greene RA, on behalf of the Perinatal Mortality Group. Perinatal Mortality in Ireland Annual Report 2011. Cork. National Perinatal Epidemiology Centre; 2013.

42. McClure EM, Bose CL, Garces A, Esamai F, Goudar SS, Patel A et al. Global network for women’s and children’s health research: a system for low-resource areas to determine probable causes of stillbirth, neonatal, and maternal death. Maternal Health, Neonatology, and Perinatology. 2015;1(11).

43. Mo-suwan L, Isaranurug S, Chanvitan P, Techasena W, Sutra S, Supakunpinyo C et al. Perinatal death pattern in the four districts of Thailand: findings from the Prospective Cohort Study of Thai Children (PCTC). J Med Assoc Thai. 2009;92(5):660-6.

44. The MRC Unit for Maternal and Infant Health Care Strategies, PPIP Users, National Department of Health. Saving Babies 2002: Third Perinatal Care Survey of South Africa. 2002.

45. Nabeel M, Bushra M, Anum Y, Muneer A, Jai K. The study of etiological and demographic characteristics of neonatal mortality and morbidity - a consecutive case series study from Pakistan. BMC Pediatr. 2012;12(131).

46. National Services Scotland. Scottish Perinatal and Infant Mortality and Morbidity Report 2011. Edinburgh; 2013.

47. Nausheen S, Soofi SB, Sadiq K, Habib A, Turab A, Memon Z et al. Validation of Verbal Autopsy Tool for Ascertaining the Causes of Stillbirth. PLoS One. 2013;8(10).

48. Nga NT, Hoa DT, Malqvist M, Persson LA, Ewald U. Causes of neonatal death: results from NeoKIP community-based trial in Quang Ninh province, Vietnam. Acta Paediatr. 2012;101(4):368-73.

49. National Institute of Population Research and Training (NIPORT), Mitra and Associates, ORC Macro. Bangladesh Demographic and Health Survey 2004. Dhaka, Bangladesh, and Calverton, Maryland, USA. National Institute of Population Research and Training, Mitra and Associates, and ORC Macro; 2005.

50. Olamijulo JA, Olaleye O. Perinatal mortality in Lagos University Teaching Hospital: a five year review. Nig Q J Hosp Med. 2011;21(4):255-61.

51. Pattinson RC, Dejong G, Theron GB. Primary Causes of Total Perinatally Related Wastage at Tygerberg-Hospital. S Afr Med J. 1989;75(2):50-3.

52. Public Health Agency of Canada. Canadian Perinatal Health Report, 2008 Edition. Ottawa, Canada; 2008.

53. Rocha R, Oliveira C, Karina Ferreira D, Bonfim C. Neonatal mortality and avoidability: an epidemiological profile analysis [Portuguese]. Revista Enfermagem UERJ. 2011;19(1):114-20.

54. Schmiegelow C, Minja D, Oesterholt M, Pehrson C, Suhrs HE, Bostrom S et al. Factors associated with and causes of perinatal mortality in northeastern Tanzania. Acta Obstet Gynecol Scand. 2012;91(9):1061-8.

55. Seaton SE, Field DJ, Draper ES, Manktelow BN, Smith GCS, Springett A et al. Socioeconomic inequalities in the rate of stillbirths by cause: A population-based study. BMJ Open. 2012;2(3).

56. Serena C, Marchetti G, Rambaldi MP, Ottanelli S, Di Tommaso M, Avagliano L et al. Stillbirth and fetal growth restriction. J Matern Fetal Neonatal Med. 2013;26(1):16-20.

57. Shah BD, Dwivedi LK. Causes of neonatal deaths among tribal women in Gujarat, India. Population Research and Policy Review. 2011;30(4):517-36. doi:<http://dx.doi.org/10.1007/s11113-010-9199-5>.

58. Simpson CD, Ye XY, Hellmann J, Tomlinson C. Trends in cause-specific mortality at a Canadian outborn NICU. Pediatrics. 2010;126(6):e1538-44.

59. Smith LK, Manktelow BN, Draper ES, Springett A, Field DJ. Nature of socioeconomic inequalities in neonatal mortality: population based study. BMJ. 2010;341(c6654).

60. The Stillbirth Collaborative Research Network Writing Group. Causes of death among stillbirths. J Am Med Assoc. 2011;306(22):2459-68.

61. Ujwala B, Alcock G, More NS, Sushmita D, Wasundhara J, Osrin D. Stillbirths and newborn deaths in slum settlements in Mumbai, India: a prospective verbal autopsy study. BMC Pregnancy Childbirth. 2012;12(39). DOI: 10.1186/1471-2393-12-39.

62. VanderWielen B, Zaleski C, Cold C, McPherson E. Wisconsin stillbirth services program: a multifocal approach to stillbirth analysis. Am J Med Genet A. 2011;155A(5):1073-80. doi:10.1002/ajmg.a.34016.

63. van Diem M, De Reu P, Eskes M, Brouwers H, Holleboom C, Slagter-Roukema T et al. National perinatal audit, a feasible initiative for the Netherlands!? A validation study. Acta Obstet Gynecol Scand. 2010;89(9):1168-73.

64. Varli IH, Petersson K, Bottinga R, Bremme K, Hofsjo A, Holm M et al. The Stockholm classification of stillbirth. Acta Obstet Gynecol Scand. 2008;87(11):1202-12. doi:10.1080/00016340802460271.

65. Wigglesworth JS. Monitoring perinatal mortality. A pathophysiological approach. Lancet. 1980;2(8196):684-6.

66. Winbo IG, Serenius FH, Dahlquist GG, Kallen BA. NICE, a new cause of death classification for stillbirths and neonatal deaths. Neonatal and Intrauterine Death Classification according to Etiology. Int J Epidemiol. 1998;27(3):499-504.

67. Winter R, Pullum T, Langston A, Mivumbi NV, Rutayisire PC, Muhoza DN et al. Trends in Neonatal Mortality in Rwanda, 2000-2010. Calverton, Maryland, USA. ICF International; 2013.

68. Wood AM, Pasupathy D, Pell JP, Fleming MS. Trends in socioeconomic inequalities in risk of sudden infant death syndrome, other causes of infant mortality, and stillbirth in Scotland: population based study. BMJ: British Medical Journal (Overseas & Retired Doctors Edition). 2012;344(7850):21-. doi:10.1136/bmj.e1552.

69. Wou K, Ouellet MP, Chen MF, Brown RN. Comparison of the aetiology of stillbirth over five decades in a single centre: A retrospective study. BMJ Open. 2014;4(6).

70. Cunningham FG, Hollier LM. Fetal death. In: Williams Obstetrics. 20th ed (Suppl 4) ed. Norwalk, Conn.: Appleton & Lange; August/September 1997.
