# Supplementary material for: Classification systems for causes of stillbirth and neonatal death, 2009–2014: an assessment of alignment with characteristics for an effective global system
Source: BMC Pregnancy Childbirth. 2016 Sep 15;16:269. doi: 10.1186/s12884-016-1040-7 (PMC5025539; doi:10.1186/s12884-016-1040-7)
Supplement: Additional file 2: — Variables used to assess system alignment with expert-identified characteristics for an effective global classification system for causes of stillbirth and neonatal death. (DOCX 57.8 kb) [file 12884_2016_1040_MOESM2_ESM.docx]

## Additional file 2

### Variables used to assess system alignment with expert-identified characteristics for an effective global classification system for causes of stillbirth and neonatal death

|  | Expert-identified characteristics for an effective global system | Variables used to assess alignment with each characteristic | Rules for assigning variable values | System is aligned with this expert-identified characteristic if variable value is… | Weight | Judgment of variable accuracy as a measure of alignment |
| --- | --- | --- | --- | --- | --- | --- |
|  | **Structural characteristics** |  |  |  |  |  |
| 1 | A global system must use rules to ensure valid assignment of cause of death categories | Does the system include a description of how causes of death are to be assigned? | - YES IF [some description of how causes should be assigned has been provided] - NO IF [otherwise, including if only the method of reaching consensus was described] | Yes | .98 | Strong |
| 2 | A global system must be able to work with all levels of data (from both low-income and high-income countries), including minimal levels |  |  | Yes for all three variables below | .98 |  |
|  |  | Regions in which used | - YES IF [used in at least one country each in LMIC and HIC] - NO IF [otherwise] |  |  | Strong |
|  |  | Was system used with verbal autopsy? | - YES IF [used with verbal autopsy] - NO IF [otherwise] |  |  | Strong |
|  |  | Countries in which used | - YES IF [used in >1 LMIC] - NO IF [otherwise] |  |  | Weak |
| 3 | A global system must ensure cause of death categories are relevant in all settings | Regions in which used | - YES IF [used in at least one country each in LMIC and HIC] - NO IF [otherwise] | Yes | .96 | Weak |
| 4 | A global system must require associated factors to be recorded and clearly distinguished from causes of death |  |  | Yes for both variables below | .94 |  |
|  |  | Allows associated factors to be recorded | - - - - YES IF [associated factors are identified as part of the system]       - NO IF [otherwise; e.g., if associated factors are identified as part of a parallel system, or if only socio-economic risk factors (e.g., mother's education), or substandard care factors, or the "three delays", or probable/possible causes are included, or if associated factors are only recorded in a statistical modelling table as confounders] |  |  | Strong |
|  |  | Distinguishes associated factors from causes of death by either having separate lists for each or including guidelines that require them to be clearly distinguished from one another | - - - - YES IF [either condition is met (separate lists or guidelines)]       - N/A IF [no associated conditions are recorded]       - NO IF [otherwise] |  |  | Strong |
| 5 | A global system must distinguish between antepartum and intrapartum conditions | Requires recording whether the stillbirth was before vs during birth (for example, does the system have categories for 'fresh' and 'macerated' stillbirth?) | - - - - YES IF [all causes are clearly either before birth or during birth]       - PARTIALLY IF [there is at least one cause that is clearly either pre-birth or during birth]       - N/A IF [system does not include stillbirth]       - NO IF [no causes clearly distinguish between deaths before and deaths during birth] | Yes | .90 | Strong |
| 6 | A global system should record the level of data available to assign the cause of death (e.g. verbal autopsy only, placental histology, autopsy, etc.) | Allows the type of data available for assigning cause of death to be recorded | - YES IF [computerized or form-based system is used and there is space for recording the type of data used (i.e., were verbal autopsy, or blood tests, or physical autopsy, or certificate of death used as a source of data for causes). Must be more than merely a data collection protocol; type of evidence should be linkable to each case.] - NO IF [otherwise, including if type of data required for the system is noted only in the Methods section of the paper but not clearly required to be recorded as part of the classification system] | Yes | .86 | Strong |
| 7 | A global system must have multiple levels of causes of death, with a small number of main categories |  |  | As indicated for the two variables below | .82 |  |
|  |  | Number of categories in top level | - Record the number as described in the paper or accompanying documents if any. If information within the paper is conflicting, note this. If there is more than one multilevel set of causes, e.g., a list for NND and a list for SB, record the relevant number for each. For analysis purposes, an average was then taken as the final value for the system. | ≤10 |  | Strong |
|  |  | Number of levels | - Record the number as described in the paper or accompanying documents if any. If there are parallel sets of causes, e.g. SB and NND, record number of levels for each (if different). For analysis purposes, an average was then taken as the final value for the system. | 2+ |  | Strong |
| 8 | A global system must include a sufficiently comprehensive list of categories to result in a low proportion of deaths classified as "other" | Maximum percent of deaths classified as "other" using this system | - YES IF [there is a category for “other” whereby the maximum percent “other” that was recorded in a use of the system in an included paper was <20%] - NO IF [system was never used, or used with a maximum percent “other” recorded in a use of the system in an included paper of 20% or greater, or if there is no category for “other”] | Yes | .80 | Weak |
|  | **Functional characteristics** |  |  |  |  |  |
| 9 | A global system must be easy to use, and produce data that are easily understood and valued by users |  |  | Yes for all four variables below | 1 |  |
|  |  | Number of deaths classified/Number of countries used in | - YES IF [the system was used to classify 500+ cases AND/OR the system was used in 2+ countries] - NO IF [otherwise] |  |  | Weak |
|  |  | Does the system include definitions for all causes of death? | - YES IF [all causes were defined in the text or accompanying documents, or if this is a modified system AND a reference to the original paper was provided AND that paper has definitions for all causes] - ONLY SOME IF [definitions were provided for just some but not all causes] - NO IF [otherwise] |  |  | Weak |
|  |  | Does the system include a description of how causes of death are to be assigned? | - YES IF [some description of how causes should be assigned was provided] - NO IF [otherwise; specifically, if only the method of reaching consensus was described] |  |  | Weak |
|  |  | Is the system used nationally? | - YES IF [system was used nationally] - NO IF [otherwise] |  |  | Weak |
| 10 | A global system must have clear guidelines for use and definitions for all terms used |  |  | Yes for both variables below | 1 |  |
|  |  | Does the system include definitions for all causes of death? | - YES IF [all causes were defined in the text or accompanying documents, or if this is a modified system AND a reference to the original paper was provided AND that paper has definitions for all causes] - ONLY SOME IF [definitions were provided for just some but not all causes] - NO IF [otherwise] |  |  | Strong |
|  |  | Does the system include a description of how causes of death are to be assigned? | - YES IF [some description of how causes should be assigned was provided] - NO IF [otherwise; specifically, if only the method of reaching consensus was described] |  |  | Strong |
| 11 | A global system must produce data that can be used to inform strategies to prevent perinatal deaths |  |  | Yes for all three variables below | .96 |  |
|  |  | Requires recording whether the stillbirth was before vs during birth (for example, does it have categories for 'fresh' and 'macerated' stillbirth?) | - - - - YES IF [all causes are clearly either before birth or during birth]       - PARTIALLY IF [there was at least one cause that is clearly either pre-birth or during birth]       - N/A IF [system does not include stillbirth] - NO IF [no causes clearly distinguish between deaths before and deaths during birth] |  |  | Weak |
|  |  | Maximum percent of deaths classified as "other" using this system | - YES IF [there was a category for “other” whereby the maximum percent “other” that was recorded in a use of the system in an included paper was <20%] - NO IF [system was never used, or used with a maximum percent “other” recorded in a use of the system in an included paper of 20% or greater, or if there is no category for “other”] |  |  | Weak |
|  |  | Is the system used nationally? | - YES IF [system was used nationally] - NO IF [otherwise] |  |  | Weak |
| 12 | A global system must require neonatal deaths to be clearly distinguished from stillbirths |  |  | Yes for both variables below | .94 |  |
|  |  | Includes guidelines that require distinguishing between SB and NND | - YES IF [guidelines or text explanation of how to distinguish SB from NND were provided] - N/A IF [system did not include both SB and NND] - NO IF [otherwise] |  |  | Strong |
|  |  | Has separate categories for SB and NND | - YES IF [every category was clearly either SB or NND] - SOME IF [only some categories were clearly either SB or NND] - N/A IF [system did not include both SB and NND] - NO IF [otherwise] |  |  | Strong |
| 13 | A global system must have high inter- and intra-rater reliability | Has system been tested for reliability? | - YES IF [formally tested for reliability, AND overall Kappa score provided, AND no Kappa <0.60] - NO IF [no formal testing was conducted within included papers, or only cause-specific Kappa scores were provided, or any overall Kappa score was below 0.60, or results were reported in the paper but were from a different paper, or otherwise] | Yes | .94 | Strong |
| 14 | A global system must be available in different formats including inexpensive ehealth and mhealth options, and in multiple languages |  |  | Yes for both variables below | .92 |  |
|  |  | Is the system available in e-format? | - YES IF [if working link to an e-format was provided in paper] - NO IF [there was a form in the paper but data could not be entered online, or link to e-format was provided but was broken, or otherwise] |  |  | Strong |
|  |  | Is the system available in more than 1 language? | - YES IF [system was found in more than one language] - NO IF [otherwise] |  |  | Weak |
| 15 | A global system must allow easy access to the data by the end-users | Does the reference paper include any guidance on how potential users might access data from the system? | - YES IF [there was a reference or link within the paper to a website that allowed the users to access the data] - UNCLEAR IF [link was provided but actual data could not be located] - NO IF [link was provided but was broken, or otherwise] | Yes | .92 | Weak |
| 16 | A global system must incorporate both stillbirths and neonatal deaths | Includes both SB and NND | - YES IF [includes both] - NO IF [includes only NND or only SB] | Yes | .86 | Strong |
| 17 | A global system must require the single most important factor leading to the death to be recorded |  |  | As indicated for the three variables below | .86 |  |
|  |  | Hierarchical | - YES IF [stated so in the text or otherwise clear from the text, or if this is a negligible modification of a system that is hierarchical] - PARTIALLY IF [e.g. one cause was stated to take precedence over all others, but remainder of causes had no hierarchy] - NO IF [stated so in the text or otherwise clear from the text, or if this is a negligible modification of a system that is not hierarchical] | No or partially |  | Weak |
|  |  | Requires that only one cause of death be recorded | - YES IF [just 1 cause was allowed (at the top level)] - UNCLEAR IF [there were separate lists of causes for SB and NND, and just 1 cause was allowed for one group, but >1 cause for the other group] - NO IF [explicitly stated in text or clear from tables that >1 cause per case was allowed] | Yes |  | Strong |
|  |  | Causes include fetal growth restriction (FGR), intrauterine growth restriction (IUGR) and SGA (small for gestational age) | - YES IF [any of these were allowed as causes at the top level of classification, including “growth restriction/placental insufficiency”] - NO IF [none of these were allowed as causes at the top level of classification; “unexplained SGA” did not count as a "yes"] | No |  | Strong |
