# Supplementary material for: Classification systems for causes of stillbirth and neonatal death, 2009–2014: an assessment of alignment with characteristics for an effective global system
Source: BMC Pregnancy Childbirth. 2016 Sep 15;16:269. doi: 10.1186/s12884-016-1040-7 (PMC5025539; doi:10.1186/s12884-016-1040-7)
Supplement: Additional file 3: — Sensitivity analyses. (DOCX 46.3 kb) [file 12884_2016_1040_MOESM3_ESM.docx]

##

## Additional file 3

### Sensitivity analyses

#### A: Sensitivity of overall alignment assessment

| How alignment was assessed | Scoring | Range | Top-ranked systems (score) |
| --- | --- | --- | --- |
| Additional file 2 rules | 1 mark for alignment with each characteristic; highest possible score 17 | (0,9) | - Froen 2009-Codac (9) - Korteweg 2006-Tulip (7) - Black 2010-CHERG, Cole 1986, Flenady 2009-PSANZ-PDC, Kotecha 2014-Wales, Ujwala 2012 (6) |
| Additional file 2 rules, weighted | As above but aligned variables are assigned weights; highest possible score 15.64 | (0,7.94) | - Froen 2009-Codac (7.94) - Korteweg 2006-Tulip (6.20) - Black 2010-CHERG, Flenady 2009-PSANZ-PDC (5.50) - Cole 1986 (5.48) - Kotecha 2014-Wales (5.42) - Ujwala 2012 (5.18) |
| Additional file 2 rules, all “weak” variables excluded | 1 mark for alignment with each characteristic with at least 1 “strong” variable; highest possible score 12 | (0,8) | - Froen 2009-Codac (8) - Korteweg 2006-Tulip (6) - Cole 1986, Flenady 2009-PSANZ-PDC, Ujwala 2012, Kidanto 2009, Schmiegelow 2012, McClure 2014-Global Network (5) |
| Additional file 2 rules, all “weak” variables excluded, weighted | As above but aligned variables are assigned weights; highest possible score 11 | (0,7.14) | - Froen 2009-Codac (7.14) - Korteweg 2006-Tulip (5.40) - McClure 2014-Global Network (4.60) - Flenady 2009-PSANZ-PDC (4.54) - Cole 1986 (4.52) - Schmiegelow 2012 (4.42) - Ujwala 2012, Kidanto 2009 (4.38) |

#### B: Sensitivity of alignment with individual characteristics to cut-offs for selected quantitative variables

| Characteristic | Variable | Range | Aligned if | Number of systems aligned |
| --- | --- | --- | --- | --- |
| 7 | - # causes in top level - # levels | - # causes: (2, 40) - # levels: (1, 4) | System has ≤10 causes in top level and 2+ levels (original cut-off) | 32 |
|  |  |  | System has ≤7 causes in top level and 2+ levels | 23 |
|  |  |  | System has ≤13 causes in top level and 2+ levels | 41 |
| 8 | % deaths classified as “other” | (0%, 68%) | System has an “other” category for which <20% of deaths have been classified as “other” (original cut-off) | 39 |
|  |  |  | System has an “other” category for which <10% of deaths have been classified as “other” | 30 |
|  |  |  | System has an “other” category for which <30% of deaths have been classified as “other” | 41 |
| 13 | Overall Kappa | (0.35, 0.93) | System has no overall Kappa <0.60 (original cut-off) | 6 |
|  |  |  | System has no overall Kappa <0.50 | 8 |
|  |  |  | System has no overall Kappa <0.70 | 4 |

Alignment with characteristic 7 was measured by the number of causes in the top level of each system and the number of levels per system. The original cut-off of ≤10 causes and 2+ levels meant that 32 of the 81 systems (40%) were aligned. Holding the number of levels at 2+ but allowing the cut-off for number of causes to vary from 7 to 13 produced a range of from 23 to 41 of the 81 systems aligned (28% to 51%).

Alignment with characteristic 8 was measured by the percent of deaths classified as “other”. The original cut-off of <20% meant that 39 of the 81 systems (48%) were aligned. Allowing this cut-off to vary from <10% to <30% produced a range of from 30 to 41 of the 81 systems aligned (37% to 51%).

Alignment with characteristic 13 was measured by overall Kappa scores. The original cut-off of no scores less than 0.60 meant that 6 of the 81 systems (7%) were aligned. Allowing this cut-off to vary from no scores less than 0.70 to no scores less than 0.50 produced a range of from 4 to 8 of 81 systems aligned (5% to 10%).
